# Engaging Parents Affected by Mental Health Problems in Pediatric and Gynecologic Practices—Implications of the KID-PROTEKT Study

**DOI:** 10.3390/children10121853

**Published:** 2023-11-26

**Authors:** Désirée Sigmund, Viola Loew, Silke Pawils

**Affiliations:** Department of Medical Psychology, University Medical Center Hamburg-Eppendorf, Martinistraße 52, 20246 Hamburg, Germany; d.sigmund@uke.de (D.S.); v.loew@uke.de (V.L.)

**Keywords:** psychosocial stress, mental illness, psychosocial assessment, psychosocial healthcare, support service access

## Abstract

Children of parents with mental illness are at higher risk of developing cognitive, mental health or physical health difficulties. Previous studies have described several barriers for reaching parents with mental health problems (MHPs) and their utilization of psychosocial services. We conducted a cluster randomized controlled study in 24 pediatric and gynecologic practices to evaluate KID-PROTEKT, a psychosocial healthcare intervention that comprises a psychosocial assessment to identify families with psychosocial needs and refer them to support services. In this paper, we analyzed whether psychosocially distressed parents with additional MHPs (identified by the PHQ-9 and GAD-7) had higher support needs, could be referred to support and utilized it in comparison to parents with psychosocial burden only. In total, 178 pregnant women and mothers with psychosocial burden were included, of whom 55 had MHPs. Participants with MHPs were distressed in their relationships more often and medical staff rated their level of support needs higher compared to parents without MHPs. There were no significant differences between the groups regarding whether they were referred to support services or utilized the recommended services. All participants were most frequently referred to family or parent counseling/care or childcare assistance. The results indicate that despite existing barriers, parents with MHPs could be reached and identified by the KID-PROTEKT psychosocial assessment. A psychosocial intervention like KID-PROTEKT can help to provide support for mentally ill parents.

## 1. Introduction

Worldwide, around one in eight adults exhibit a mental illness [1]. In Germany, the twelve-month prevalence of mental illness among adults has been reported as 27.7% [2]. According to a birth cohort study in Sweden, around 11% of children currently live with a mentally ill parent [3]. A US survey which collected data between the years 2008 and 2014 reported that 18.2% of parents had some mental illness, while 3.8% of parents had a serious mental illness [4]. These statistics indicate that a substantial proportion of children grow up in a family where at least one parent has a mental disorder.

Children of mentally ill parents have a higher risk of a range of impaired outcomes, such as higher levels of internalizing and externalizing behavior [5] or poorer developmental outcomes [6]. Furthermore, there is a strong relationship between parent mental health and child mental health: children of parents with a mental illness are more likely to develop a mental health psychiatric disorder in childhood, adolescence or adulthood compared to the general population [7]. The BELLA study is a large longitudinal study which investigates mental health- and quality-of-life-associated factors and the utilization of mental health services by children, adolescents and their families in Germany. The findings of the study suggest that the risk of having a mental disorder in general is twice as high for children of mentally ill parents [8]. Children seem to be affected even more if both parents have a mental illness [9].

Mental illness in the perinatal period has a high relevance, as maternal stress can exert an influence on the development of the fetus due to mechanisms like fetal programming and epigenetics [10]. For instance, maternal perinatal illness has been associated with adverse obstetric outcomes like preterm birth [11,12] and low birthweight [13]. Infants are a vulnerable group, as a child’s first year of life is crucial for their biological, emotional and cognitive development [14]. In summary, parental mental health can exert a significant influence on children that can last up to adulthood.

Next to genetic and biologic factors, environmental factors such as family functioning play an essential role in the development of mental disorders in children [15]. Parents with mental illness may be less sensitive and show less positive affect to their children [16], which could potentially have an impact on parent–child interactions. Psychosocial risks such as socioeconomic disadvantages, unemployment and partner violence [17,18] are related to parental mental health, but can also contribute to the risk of adverse consequences on child health and developmental outcomes [19].

Mental health and psychosocial services are a source of support for parents with mental health issues and can moderate the effect of parental mental health on children [20]. Next to adult mental health interventions, family-centered interventions have proven effective in improving psychopathologic symptoms in children [21]. Furthermore, early preventive interventions for parents with mental illness and their children are important to ensure a healthy child development. However, reaching parents with mental illness remains a challenge [22]. Despite the existence of healthcare services, it has been shown that less than 50% of those with a mental illness engage in mental health services [2,23]. A German survey of 844 parents reported that around 30% of mentally ill parents utilized mental health services and 12% engaged in family-centered support services [22].

Several barriers for the utilization of psychosocial services have been reported. A systematic review by Sambrook Smith et al. [24] identified several aspects that hindered engagement in mental health services during the perinatal period. These included individual factors such as a lack of awareness (both among women and healthcare professionals) and negative attitudes towards mental illness, including fear of stigma. Regarding organizational factors, role conflicts were described. Lastly, structural and sociocultural factors such as language barriers were found [24]. Among healthcare professionals, barriers such as lack of time and lack of resources to conduct a psychosocial or mental health screening can prevent the identification of needs [25]. A German study revealed that next to the stigma of mental illness and fears of losing custody, the perception that the child’s needs were prioritized over the mother’s was seen as a barrier. Other limitations for the utilization of services among parents with mental health disorders include a lack of knowledge about these services [26].

The assessment of parental support needs can help to initiate interventions and promote the wellbeing of parents and their children. In gynecology, several guidelines recommend including a psychosocial screening in routine perinatal care [27]. Various instruments and models [28,29] to address mental health in obstetric healthcare have been developed. Also, in pediatrics, increasingly family-centered care is being prioritized [30] and the importance of addressing the mental health of parents has been recognized [20,31]. A facilitating factor that can be helpful in addressing mental health problems by healthcare professionals is a caring and interested attitude [32]. From the parents’ perspective, trust in the healthcare provider is important [33]. However, psychosocial assessment in the gynecologic and pediatric setting remains a challenge [34]. Overall, families with parental mental illness are a specific and vulnerable group that should be considered in healthcare areas such as pediatrics and gynecology.

The KID-PROTEKT project was executed from September 2019 to February 2021 in gynecologic and pediatric practices in Germany to investigate the effectiveness of a psychosocial healthcare intervention for families in the perinatal period. The key component of KID-PROTEKT is a psychosocial assessment consisting of a psychosocial screening and an interview with healthcare staff (physicians, nurses). The goal of KID-PROTEKT is to promote the identification of psychosocially burdened families in the perinatal period and to provide fitting referrals to support services for these families. Around 8500 pregnant women and families with infants were enrolled in the main study.

Since parents with mental health problems (MHPs) have been identified as a specific group with several factors hindering their utilization of support, we wanted to investigate this specific group within the KID-PROTEKT study in more detail. The goal of this sub-study was to explore a) whether parents with symptoms of mental illness were identified as having a higher level of support needs, b) were more likely to be referred to a support service than parents with psychosocial burden only and c) what kind of recommendations for services they received.

## 2. Materials and Methods

This sub-study was part of a multicenter, randomized controlled trial on the effectiveness of KID-PROTEKT conducted in 24 gynecologic (*n* = 13) and pediatric (*n* = 11) medical practices in Hamburg, Germany, and surrounding rural areas. The results of the main trial are reported in a separate paper [35]. The present paper comprises findings on secondary outcomes in a sub-sample of the main trial. Ethical approval was obtained from the Ethics Committee of the Medical Association Hamburg (PV 6027) and the Local Ethics Committee at the Center of Psychosocial Medicine (LPEK-0119).

### 2.1. Participants

Pregnant women, women in childbed and parents with infants (first year of life) with psychosocial burden were included in this study. All families who attended the gynecologic and pediatric practice for a routine examination were invited to participate in KID-PROTEKT. In gynecologic practices, pregnant women were recruited during pregnancy (first to third trimester) or during postpartum follow-up care (6–8 weeks after birth). In pediatric practices, all parents attending regular well-child visits (German “U-Untersuchungen”) ranging from three to ten days postnatal (“U2”) up to the child’s first year of life (“U6”) were invited to participate.

In the main study, data of 7952 participants could be analyzed, of whicg. 1556 participants with psychosocial burden (*n* = 1046 from pediatric and *n* = 510 from gynecologic practices) underwent the whole psychosocial assessment. Among those, 45% gave additional consent to take part in the telephone interview and 286 could be reached and were invited to fill out the online survey. Finally, 182 participants responded to the online survey. Since only 4 fathers participated, they were excluded, resulting in a sample of 178 participants in this sub-study. In the following sections, participants will also be referred to as parents, although only mothers participated.

### 2.2. KID-PROTEKT

**Figure 1:**
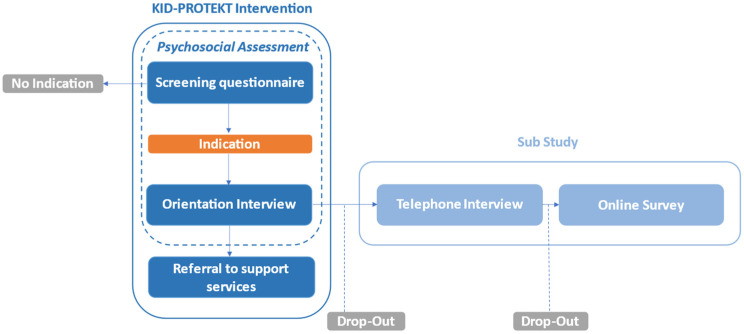
KID-PROTEKT intervention and study flow chart.

KID-PROTEKT is a child-centered psychosocial healthcare intervention that comprises a psychosocial assessment conducted by pediatric and gynecologic healthcare professionals (e.g., physicians, nurses). This psychosocial assessment intends to identify families with psychosocial stress and explore their specific needs (psychological, social or medical) in a brief conversation. Finally, families can be referred to the appropriate support services (e.g., counseling services). The design of KID-PROTEKT and the procedure of this sub-study are illustrated in Figure 1. Before the study, healthcare professionals received training that comprised different topics, such as information on early childhood interventions and psychosocial stress during the perinatal period. Healthcare providers learnt how to evaluate families’ psychosocial situations and how to refer them to appropriate support services.

The psychosocial assessment was conducted in two steps. Firstly, a screening on psychosocial stress was handed out. If the screening was returned without an indication of psychosocial stress, no further steps were taken. If families reported at least one area of psychosocial stress, an orientation interview was offered by healthcare professionals. During the orientation interview, the psychosocial stressors resulting from the screening were explored and the families’ degree of support needs was rated. If support needs were identified, staff informed families about support services, provided contact information for regional support services or organized an appointment. While in half of the practices, the psychosocial assessment and referral of families with psychosocial needs was conducted by healthcare providers alone, in 7 practices, a social worker was additionally integrated to function as a baby pilot. The baby pilots supported and supervised healthcare providers and acted as a support service by providing a regular counseling hour within the practice.

### 2.3. Instruments

#### 2.3.1. Psychosocial Assessment

The psychosocial assessment in KID-PROTEKT comprised a screening questionnaire on psychosocial stress and an orientation interview. The KID-PROTEKT stress questionnaire was developed for this study based on experiences from a previous project (the “Babylotse” family intervention) [36]. It was designed as an eleven-item self-report questionnaire to measure different areas of psychosocial stress. Three items measured sociodemographic characteristics such as the type of examination and age in categories (e.g., 18 to 21). The other items were answered dichotomously (yes/no). One item applied to symptoms such as loss of joy, tiredness or having a stressful experience and one item assessed being a single parent. In the six remaining items, families were asked to report whether they felt distressed in the following areas: (1) coping with everyday life, (2) relationship, (3) life situation, (4) pregnancy and birth, (5) children and education, (6) other stress factors. If there was at least one psychosocial stressor present in the questionnaire, an orientation interview was initiated. The orientation interview conducted by healthcare providers was documented on a semi-structured sheet that contained basic information of the interview such as date, duration and interviewer (nurse or physician). The discussed stressors were recorded analogously to the categories in the screening questionnaire. The level of support needs were rated by healthcare providers on a 6-option scale from “no needs” to “very high needs”. Finally, it was registered whether the family was referred to a support service as well as the kind of referral (handing out information about support services or linking families directly, such as making an appointment) and the recommended institutions.

#### 2.3.2. Sociodemographic Data and Utilization of Support Services

Sociodemographic information and the utilization of support services by participants were assessed during the telephone interviews. Participants were asked several questions regarding their age, gender, relationship, family structure, education and citizenship. Those who received a referral to a support service were asked whether they utilized the service and how satisfied they were with it.

#### 2.3.3. Depression and Anxiety Symptoms

Symptoms of depression and anxiety were assessed using the Patient Health Questionnaire (PHQ-9) [37] and the Generalized Anxiety Disorder Scale (GAD-7) [38]. Both are structured screening questionnaires based on the criteria for depression and generalized anxiety disorder, respectively, of the Diagnostic and Statistical Manual of Mental Disorders (DSM-5) [39] Participants are asked to rate whether or how often they experienced symptoms within the last two weeks on a 4-point Likert scale. The items are added to a sum score and can be used to categorize the severity of the symptoms (minimal, mild, moderate or moderately severe).

### 2.4. Procedure

All pregnant women and families were informed about the study and invited to participate before their appointment for a routine pregnancy check-up or well-child visit. If they agreed, they filled out the screening questionnaire on psychosocial stress in the waiting room. Subsequently, the questionnaire was evaluated by the practice staff. Families who reported psychosocial burden were offered an orientation interview that was conducted either immediately at the practice or during the following days via telephone. After the orientation interview, participants received a study information sheet and gave their informed consent. All documents (screening questionnaire, documentation of orientation interview) were safely put in a letterbox and picked up by a study nurse from the research team.

Pregnant women and families who consented were contacted to take part in the telephone interview and online survey. The interview was performed by members of the research team. At the end of the interview, the participants received the link to the online survey. The online survey was conducted on the platform SoSci-survey (https://www.soscisurvey.de/, accessed on 1 February 2020). All participants who took part in the telephone interview and the online survey received a gift voucher of EUR 5 per person. Upon completion of the online survey, participants were sent the gift voucher via post.

### 2.5. Data Analysis

Data analyses were performed with the program SPSS (Version 29, IBM Corp) [40]. Participants with a screening questionnaire in which more than three items were missing could not be included in the analysis. The items of the PHQ-9 and GAD-7 were added up to a sum score. A cutoff of 10 in the PHQ-9 and GAD-7 demonstrated good sensitivity and was therefore used to classify participants with psychosocial burden plus mental health problems (MHPs) [41]. Descriptive statistics were used to investigate the demographic and psychosocial characteristics of the sample as recorded during the psychosocial assessment, the number of patients referred to support services and the answers to the telephone interviews and online survey. For categorical variables, frequencies and percentages were used, whereas for continuous variables, means and standard deviations were used. To test differences in frequencies between the groups, we used χ^2^ tests. Differences in metrical variables between the two groups were analyzed by *t*-tests. The recommended institutions participants were referred to were categorized using the software MAXQDA (Version 2022) [42].

## 3. Results

### 3.1. Sample

Fifty-one (28.7%) participants had an elevated PHQ-9 score and twenty-seven (15.2%) had a GAD-7 score of 10 or higher. Combining both measures, in total, 55 (30.9%) had either a high PHQ-9 or GAD-7 score and were added to the group of parents with psychosocial burden plus MHPs. The group of parents with solely psychosocial burden consisted of 123 (69.1%) participants.

Sociodemographic variables are displayed in Table 1. All participants were female. Most parents were in a relationship with their child’s other parent (87–91.9%) and had been born in Germany (81.8–84.6%). Among participants with MHPs, 14.0%, and 8.1% of participants in the other group were reported to be single parents. The majority in both groups had graduated high school (German “Abitur”; 61.8–70.7%) and reported to be on maternity or parental leave (61.8–67.5%). Participants with MHPs were slightly younger (M= 31.58, SD = 4.30) than participants in the other group (M = 32.98, SD = 4.32), t(176) = 2.003, *p* = 0.047, Cohen’s d = 0.325. There was also a significant difference in terms of professional and academic education between the two groups, χ^2^(1, 178) = 19.432, *p* = 0.013, Cramer V = 0.330. For other sociodemographic variables, no significant differences were detected.

The 55 participants with MHPs presented, on average, a PHQ-9 score of 12.40 (SD = 2.83, Range 6–20) and a GAD-7 score of 9.76 (SD = 4.13 Range 3–19). The remaining 123 participants were below 10 at baseline and had, on average, a PHD-9 score of 5.36 (SD = 2.29, Range 0–9) and a GAD-7 score of 3.96 (SD = 2.40, Range 0–9). There were no significant differences between questionnaire timings (trimester/well-child visit) and PHQ-9 and GAD-7 scores.

### 3.2. Identification and Referral of Parents with Psychological Symptoms

The duration of the orientation interview was similar in both groups (MHP: M = 8.78, SD = 4.20; other: M = 8.70, SD = 5.20) and did not differ significantly, t(170) = −0.104, *p* = 0.917. Table 2 presents the psychosocial risks discussed during the orientation interview for both groups. In both groups, being distressed in daily life due to tiredness, loneliness, mental or somatic disorders was the most common psychosocial stressor discussed with medical staff (38.2% and 33.6%). Parents with MHPs reported to be distressed in their partnerships more often (21.8%) than parents in the other group (9.0%), χ^2^(1, 177) = 5.495, *p* = 0.019. For the other stressors, there were no significant differences in frequency between both groups.

There was a significant difference in the level of support needs between the groups, t(173) = −2.335, *p* = 0.021, Cohen’s d = −0.382. The level of support needs (range 1–6) rated by healthcare professionals was higher in parents with MHPs (M = 3.50, SD = 1.67) compared to the other group (M = 2.84, SD = 1.67).

Next, we investigated how many participants were referred. In the group of parents with solely psychosocial burden, 38 (31.9%) were referred, while 81 (68.1%) were not. For four participants, information on referral was missing. Among parents with MHPs, twenty-three (42.6%) were referred, while thirty-one (57.4%) were not (one missing). There was no significant difference between the two groups regarding referrals, χ^2^(1, 173) = 1.849, *p* = 0.174.

The recommended support services are displayed in Table 3. Referrals were made to a wide range of support services that included counseling, care services, support for social needs (e.g., financial support) and midwives. In both groups, referrals to counseling services such as parenting counseling or family counseling were the most frequent. Furthermore, childcare services were often recommended. Mental health services were recommended in both groups. A quarter (23.8%) of parents with MHPs received a referral to mental health services such as psychotherapy. More parents with MHPs (66.7%) were referred to a baby pilot than parents in the other group (34.2%).

Among parents with MHPs who received a referral, five (21.7%) utilized at least one of the referred services. Among parents with psychosocial burden alone, 11 (29.7%) utilized the services. There was no significant difference between the groups, χ^2^(1, 60) = 0.563, *p* = 0.496.

## 4. Discussion

Considering the adverse consequences of parental mental illness for children, it is important to identify the support needs of parents in the perinatal period and provide interventions for the whole family. However, parents with mental illness are difficult to reach since only a limited number of people with mental illness utilize mental healthcare services [2,23]. KID-PROTEKT is a psychosocial healthcare intervention for gynecologic and pediatric settings to identify families with psychosocial risks and refer them to adequate support services. By addressing psychosocial and mental health problems in these healthcare settings, parents can receive adequate support early on.

This sub-study focused on the group of parents with MHPs within the KID-PROTEKT study, which tested the effectiveness of KID-PROTEKT in gynecologic and pediatric practices. We investigated whether parents with MHPs could be identified and referred in the same way as parents with solely psychosocial burden. Furthermore, we analyzed what kind of recommendations parents with MHPs received. The results show that both groups of parents experienced psychosocial stressors to a similar degree, except for partnership problems, which were more prevalent in parents with MHPs. During the orientation interview, parents with MHPs were rated as having a higher level of support needs than parents with only psychosocial stressors. This shows that healthcare professionals were able to identify parents with MHPs and recognize their higher support needs during the psychosocial assessment.

There was no significant difference in the frequency of referrals between the groups. These results indicate that parents with MHPs could be referred to services and utilized them to a similar degree as parents with other psychosocial burden. Regarding the type of institutions recommended, we found that referrals were very specific and adjusted to the support needs and living area of participants. In both groups, some parents received a referral to mental health services such as a psychotherapist. In the group of parents with MHPs, 23.8% were referred to mental health services, compared to 13.2% in the other group. In general, most parents received counseling in relation to pregnancy, children or parenting as well as childcare services. While these are important services to support distressed parents in their daily life, in the case of mental illness, a referral to a mental health practitioner for diagnostics and further intervention is important. The reason that fewer referrals to mental health services were recorded could be various. Referrals to mental health services could have been challenging for healthcare professionals at the practices we studied, because many participants presented with more than one problem. The inability to provide fitting recommendations for mental health services, due to lacking referral options for example, has been documented as a barrier in other studies as well [33,43]. The finding that more parents with MHPs were referred to baby pilots suggests that in some cases, healthcare professionals were unable to provide referrals to mental health services themselves due to the complexity of the families’ needs. Also, the project focused on very different psychosocial risks, and not only mental illness, so referrals were very broad. Healthcare professionals were educated beforehand and received information and lists of regional support services, but there was no manual that indicated specific processes for referrals. Furthermore, addressing mental illness, in contrast to other psychosocial stressors, could more challenging since parents might feel stigmatized [44] and healthcare staff might feel insecure about how to address mental health [45]. Previous studies revealed several barriers for addressing mental health during appointments on the part of professionals, such as a lack of experience and knowledge about mental health assessments [24,45]. Thus, there remains a need for training healthcare professionals on how to conduct psychosocial assessments [25]. While in all healthcare professionals who took part in KID-PROTEKT received training on psychosocial aspects and childcare services, further specialized training on mental illness in the perinatal period might be required.

We found that around a quarter of participants in both groups utilized the recommended services. This approximately corresponds to the percentage of the whole sample in the main study [35]. A German study found that 30% of mentally ill parents engaged in psychotherapy and 12% in family services [22], whereas a US representative study revealed a utilization rate of 40% in the general population [23]. The lower utilization rate in parents is expectable due to special barriers for the utilization of support such as fear of stigma and guilt. Strengthening the collaboration between gynecologic and pediatric healthcare providers, mental health services and child and youth welfare is important in order to reduce structural barriers and to help patients get involved in support services more easily. To accomplish better collaboration, more networking is required, which could be promoted by regular networking meetings.

The following limitations of the present study should be considered. Firstly, the outcomes of this study were secondary and thus only investigated in a smaller sub-sample. Therefore, this study probably has a selection bias as only participants who agreed to the interview and online survey and were reached could be included. Pregnant women and parents who were especially burdened might have refused to take part in the additional telephone call and online survey and may not be represented in this sample. In addition, participants who were unable to speak or read German could not be included in this sub-study as knowledge of German was required. Furthermore, the distribution of mothers and fathers was very uneven and only mothers were included in this sample. It is a known problem in studies on the perinatal period that fathers are underrepresented [46]. As a result, the sample in this sub-study was rather small in contrast to the whole KID-PROTEKT sample and the external validity of this study might be limited. Therefore, caution is required when applying the findings to other settings or populations. Future studies should aim to include fathers, as their perspective is just as important and would generate new insights. Lastly, we defined parental psychopathology by two instruments, PHQ-9 and GAD-7, which measure symptoms of depression and anxiety. Although depression and anxiety are two of the most prevalent mental health disorders in general, we did not cover all symptoms of mental illness.

## 5. Conclusions

The results of our study revealed positive effects of KID-PROTEKT for families in the perinatal period. By implementing a psychosocial assessment into pediatric and gynecologic practices, participants with psychosocial burden were identified and referred to support services. Parents with additional symptoms of mental illness were connected to services as often as parents without mental illness. The psychosocial assessment enabled a structural identification of psychosocial needs that provided a basis for referrals to support services. The training that took part beforehand helped to equip healthcare professionals with the competence and confidence to address psychosocial needs and organize referrals to further support. While many of the participants were connected to services, mental health services were not recommended frequently. More specialized training on parental mental health is required to prepare healthcare providers better for this vulnerable group.

## Figures and Tables

**Table 1:** Demographic variables of all participants (*N* = 178).

	Participants with Psychosocial Burden Plus MHPs n (%)	Participants with Psychosocial Burden n (%)	*p*
Age	M = 31.58 (SD = 4.30), Range 22–40	M = 32.98 (SD = 4.32), Range 20–45	0.047
Household members	M = 3.22 (SD = 0.94), Range 1–6	M = 3.10 (SD = 0.96), Range 1–6	0.437
Participation			
Pediatrics	36 (65.5)	67 (54.5)	
Gynecology	19 (34.5)	56 (45.4)	
Partnership			0.593
Single	6 (11.1)	8 (6.5)	
In relationship with other parent	47 (87.0)	113 (91.9)	
In relationship with different partner	1 (1.9)	1 (0.8)	
Single parent	8 (14.8)	10 (8.2)	0.335
Living in Germany			0.648
Since birth	45 (81.8)	104 (84.6)	
Immigrated	10 (18.2)	19 (15.4)	
School education			0.651
Did not graduate	0 (0)	1 (0.8)	
High school graduation after 9th grade	4 (7.3)	5 (4.1)	
High school graduation after 10th grade	16 (29.1)	29 (23.6)	
High school graduation (Abitur)	34 (61.8)	87 (70.7)	
Other	1 (1.8)	1 (0.8)	
Job or academic education			0.013
No education	3 (5.5)	4 (3.3)	
Vocational training	5 (9.1)	18 (14.6.)	
Educational job training	25 (45.4)	30 (24.4)	
Graduation from professional school	2 (3.6)	2 (1.6)	
Graduation from university of applied science/university/college	18 (32.7)	63 (51.2)	
Other	1 (1.8)	6 (4.9)	
Occupation			
Not employed	6 (10.9)	7 (5.7)	0.175
Part-time	8 (14.5)	8 (6.5)	
Full-time	5 (9.1)	22 (17.9)	
Maternity/parental leave, other leave of absence	34 (61.8)	83 (67.5)	
Other	2 (3.6)	3 (2.4)	

**Table 2:** Psychosocial stressors in orientation interview.

Burdened in…	Participants with Psychosocial Burden Plus MHPs n (%)	Participants with Psychosocial Burden n (%)	*p*
Daily life	21 (38.2)	41 (33.6)	0.555
Partnership	12 (21.8)	11 (9.0)	0.019
Living conditions	18 (32.7)	41 (33.6)	0.901
Pregnancy and birth	19 (34.5)	38 (31.1)	0.654
Children and parenting	12 (21.8)	17 (13.9)	0.190
Other	17 (30.9)	39 (32)	0.889

**Table 3:** Frequency of recommended support services.

Support Service	Parents with Psychosocial Burden Plus MHPs * n (%)	Parents with Psychosocial Burden n (%)
Educational counseling	5 (23.8)	3 (7.9)
Family counseling and resource centers	4 (19)	13 (34.2)
Parent counseling and support for children aged 0–3	5 (23.8)	11 (28.9)
Other counseling services	6 (28.6)	5 (13.2)
Childcare services	6 (28.6)	7 (18.4)
Services for social-economic problems	2 (9.5)	9 (23.7)
Single parent meetings	2 (9.5)	3 (7.9)
Mental health services	5 (23.8)	5 (13.2)
Midwife	2 (9.5)	2 (5.3)
Colic clinic	3 (14.3)	1 (2.6)
Websites and information material	0 (0)	5 (13.2)
Healthcare services	1 (4.8)	4 (10.5)
Other recommendations	2 (9.5)	5 (13.2)
Baby pilots	14 (66.7)	13 (34.2)

* For 3 participants, the service recommended was not documented.

## Data Availability

Data are available upon reasonable request to the corresponding author and after consultation of the local data protection manager. The data are not publicly available due to privacy and ethical restrictions.
